# Restrictive Lung Function Patterns and Sex Differences in Primary School Children Exposed to PM2.5 in Chiang Mai, Northern Thailand

**DOI:** 10.3390/ijerph22101530

**Published:** 2025-10-06

**Authors:** Pakaphorn Ngamsang, Anurak Wongta, Sawaeng Kawichai, Natthapol Kosashunhanan, Hataichanok Chuljerm, Wiritphon Khiaolaongam, Praporn Kijkuokool, Putita Jiraya, Puriwat Fakfum, Wason Parklak, Kanokwan Kulprachakarn

**Affiliations:** 1 School of Health Sciences Research, Research Institute for Health Sciences, Chiang Mai University, Chiang Mai 50200, Thailand; pakaporn_ng@cmu.ac.th (P.N.); anurak.wongta@cmu.ac.th (A.W.); natthapol.ko@cmu.ac.th (N.K.); hataichanok.ch@cmu.ac.th (H.C.); wiritphon_k@cmu.ac.th (W.K.); praporn_k@cmu.ac.th (P.K.); puriwat_f@cmu.ac.th (P.F.); 2 Research Center for Non-Infectious Diseases and Environmental Health, Research Institute for Health Sciences, Chiang Mai University, Chiang Mai 50200, Thailand; sawaeng.kaw@cmu.ac.th (S.K.); putita_jiraya@cmu.ac.th (P.J.)

**Keywords:** air pollution, PM2.5, biomass burning, indoor air pollution, children, restrictive, spirometry, GLI 2012, sex characteristics, Thailand

## Abstract

Northern Thailand experiences annual haze events with fine particulate matter (PM2.5) exceeding standards, posing risks to schoolchildren. This cross-sectional study (Chiang Mai, 2024) evaluated respiratory impacts among primary school children aged 8–12 years. Daily mean PM2.5 concentrations were obtained from a single fixed-site monitoring station (36T) located within 2 km of the spirometry site. Among 93 children with acceptable spirometry, 52% exhibited restrictive, 18% obstructive, and 30% had normal function. After adjustment for BMI, males had significantly lower odds of any pulmonary abnormality than females (AOR = 0.084; 95% CI 0.017–0.417; *p* = 0.002). The mean FEV_1_/FVC ratio was normal (86.30 ± 13.07%), whereas mean FVC, FEV_1_, and PEF were significantly below predicted values, indicating a predominantly restrictive pattern. This predominance likely reflects cumulative exposure to biomass-burning related PM2.5 during the haze season, infiltration of outdoor PM2.5 into indoor environments alongside indoor sources, and the vulnerability of developing lungs in children’s factors that reduce lung volumes while largely preserving the FEV_1_/FVC ratio. The exposure assessment provides pragmatic, proximity-based estimates but is limited by reliance on one station and one season, which may not capture spatial or temporal variability. These findings highlight sex-based susceptibility and support stronger air quality protections for children.

## 1. Introduction

Air pollution is a major global public health concern, linked to a wide range of negative health outcomes such as respiratory and cardiovascular diseases, and increased mortality rates [1]. One of the most harmful pollutants is fine particulate matter with a diameter of less than 2.5 micrometers (PM2.5). Due to its small size, PM2.5 can penetrate deep into the lungs and enter the bloodstream, where it can trigger inflammation and oxidative stress [2]. These biological responses can damage tissues, impair lung function, and contribute to serious health effects, especially in children whose lungs and immune systems are still developing [3]. In Thailand, the upper northern region is particularly affected by high levels of PM2.5. Chiang Mai Province is one of the areas most severely impacted, with PM2.5 concentrations frequently exceeding both national and WHO air quality standards [1]. A study highlighted Chiang Mai as one of the most polluted cities in the world [4]. For example, in March 2021, the daily average PM2.5 concentration peaked at 187.6 µg/m^3^, far above the safe limits. This spike was associated with increased hospital admissions and significant health risks across all age groups [4]. Mueang Chiang Mai District is particularly vulnerable due to a variety of PM2.5 sources, including traffic emissions, construction activities, seasonal biomass burning, and forest fires. Unlike other districts in the province that are mainly affected by agricultural burning, the city also faces urban pollution and has the highest population density in the region. Seasonal variations in air quality create serious health risks for local residents, particularly for vulnerable groups such as children.

Previous studies have shown that high PM2.5 exposure is closely linked to respiratory problems in children [5]. Children are especially vulnerable due to several physiological and behavioral factors: they breathe more frequently than adults, spend more time outdoors, and have a higher oxygen demand relative to body weight. In addition, their airways are narrower, their breathing zone is closer to the ground where pollutants concentrate, and their detoxification systems are not yet fully developed [6,7]. These combined factors increase children’s susceptibility to air pollution and its health impacts.

Numerous studies have shown that pulmonary function serves as an effective indicator of early-stage lung disease [8]. PM2.5 can impair lung function through airway–parenchymal inflammation, oxidative stress, and disrupted lung growth, leading to reduced lung volumes and abnormal spirometry in children whose lungs are still developing. Longitudinal studies across Asia, Europe, and China consistently link chronic PM2.5 exposure to progressive declines in lung function; for example, the Children’s Health Study reported slower growth in FVC and FEV_1_ with long-term exposure [9,10,11,12]. These findings support using pulmonary function tests, particularly spirometry, to monitor both short- and long-term effects of air pollution on pediatric respiratory health. Consistent with public-health guidance, children should limit outdoor activity on days when particulate levels exceed standards, as such exceedances are associated with increased respiratory morbidity [13,14].

Therefore, this study aims to assess the concentration levels of PM2.5 and its impact on the respiratory health of school-aged children in Chiang Mai, Thailand. Lung function was measured using spirometry, focusing on key indicators including Forced Vital Capacity (FVC), Forced Expiratory Volume in one second (FEV_1_), and Peak Expiratory Flow (PEF) [15]. The spirometry tests were conducted in Mueang Chiang Mai District, where participants had lived in high-exposure areas for several years and were regularly exposed to elevated PM2.5 levels, especially during the annual haze season. Although the tests were performed in July, a period outside the peak pollution season, the significantly high PM2.5 levels observed in preceding months particularly in March and April suggest that the lung function impairments detected may reflect cumulative or residual effects of long-term exposure. This research provides a scientific basis for understanding the harmful effects of PM2.5 on children’s respiratory health. The 8–12-year age group was selected because it represents a critical stage of lung development, during which air pollution exposure may lead to long-term respiratory and cardiovascular effects. Children in this age group are also more active outdoors, increasing their exposure to ambient pollutants. Moreover, they are typically able to understand and follow clinical instructions, such as those required for spirometry, allowing for more reliable data collection. Epidemiological evidence also indicates that the association between particulate matter exposure and reduced lung function tends to be more pronounced in this age group than in older children. Although some health-related evidence exists, studies specifically focusing on school-aged children living in urban Chiang Mai an area with dense traffic and recurring seasonal haze remain limited [16]. An unusual predominance of restrictive over obstructive spirometry patterns has been observed in primary school children, with a preserved FEV_1_/FVC ratio indicating reduced lung volumes rather than airway obstruction. Addressing this research gap is essential for informing effective public health strategies to protect this vulnerable population.

## 2. Materials and Methods

This cross-sectional field study began in July 2024, following standard research protocols. Ethical approval was obtained from the Human Experimentation Committee of the Research Institute for Health Sciences (RIHES), Chiang Mai University, Thailand (Project No. 4/67; 25 July 2024). Permission to conduct the study in public primary schools was granted by the Director of Education. This was a cross-sectional study in which exposures and outcomes were measured at a single time point, precluding causal inference. Nonetheless, the findings provide important preliminary evidence to inform future longitudinal studies aimed at establishing temporality and evaluating potential long-term effects.

### 2.1. Study Design

**Figure 1:**
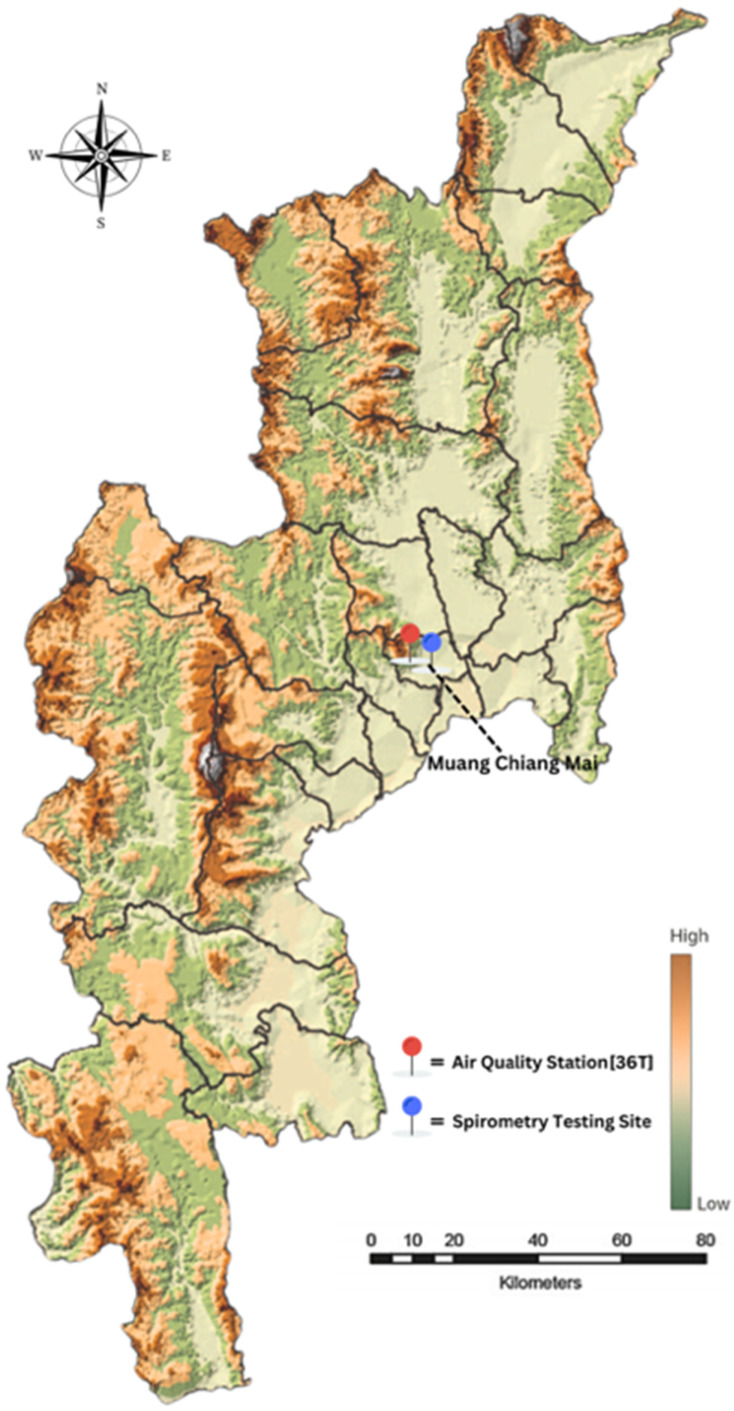
Study area and monitoring site in Chiang Mai Province, Thailand.

This study was conducted in Chiang Mai Province, northern Thailand, an area predominantly characterized by mountainous terrain. Figure 1 illustrates the study area and monitoring site in Chiang Mai Province, Thailand. The background color gradient indicates relative elevation, with darker brown shades representing higher topography. The red marker denotes the air quality monitoring station (36T), located at Yupparaj Wittayalai School, while the blue marker indicates the spirometry testing site at Wat Chiang Yuen Municipal School. Both selected locations are situated in Mueang Chiang Mai District and are within a 2-km radius of each other. Participants included primary school students aged 8–12 years, who underwent pulmonary function testing using spirometry. A total of 110 students were randomly selected across all grade levels. The spirometry tests were conducted in July 2024, when ambient PM2.5 levels in Chiang Mai are typically low, to ensure the availability of volunteers and their optimal physical condition. By that time, participants were more likely to have recovered from seasonal respiratory illnesses, such as influenza or colds caused by dry air, which typically occurred during the early months of the year [17,18]. Since spirometry testing required participants to be free from symptoms such as coughing, sneezing, or a runny nose, the presence of these symptoms could have resulted in inaccurate measurements and failure to meet standard procedural guidelines [19]. Additionally, July was selected during the school semester when students were present and accessible, reducing both practical and ethical concerns. Conducting tests during the peak pollution months (March–April) would have posed ethical challenges, as children might have already experienced respiratory symptoms, and testing could have increased discomfort or exacerbated existing conditions. Furthermore, conducting the tests during the low-exposure season minimized acute confounding effects from active pollution events and provided a clearer baseline of lung function that may have reflected underlying chronic or residual impairments. Although this timing limited the direct observation of acute exposure effects, previous studies [20] suggested that repeated seasonal exposure to air pollution could have contributed to chronic or subacute respiratory impacts detectable beyond the exposure period. Given that children in Chiang Mai were repeatedly exposed to high levels of PM2.5 on an annual basis, the lung function results collected in July might have reflected cumulative or residual effects, even in the absence of acute pollution during the testing period. Therefore, the findings in this study should be interpreted as inferences of cumulative and/or residual exposure rather than acute effects. The lung function impairments observed were likely shaped by prior repeated exposures to elevated PM2.5 levels during previous haze seasons, not by pollution occurring on or near the day of spirometry testing.

From Table 1, the annual and daily average concentrations of PM2.5 in Chiang Mai Province consistently exceed both the national standards of Thailand and the air quality guidelines established by the WHO. Specifically, the annual average PM2.5 concentration in Chiang Mai is 27.6 µg/m^3^, which is higher than Thailand’s annual standard of 25 µg/m^3^, and more than five times the WHO’s 2021 guideline of 5 µg/m^3^. In terms of daily exposure, the Thai standard sets the 24-h average limit at 37.5 µg/m^3^, while the WHO’s 2021 updated guideline is much stricter, at 15 µg/m^3^ [5]. As shown in Table 1, during the smog season (particularly March and April), daily PM2.5 concentrations frequently exceeded both thresholds, with some days surpassing 150 µg/m^3^. This persistent and repeated exposure to high PM2.5 levels both in short-term spikes and long-term averages poses significant risks to respiratory health, particularly among vulnerable populations such as children.

### 2.2. Calculating Cumulative PM2.5 Exposure (AUC)

Cumulative exposure to PM2.5 was estimated using the area under the curve (AUC) method, calculated over a 120-day period preceding the lung function assessment. The trapezoidal rule, a standard numerical integration technique, was applied to daily PM2.5 concentration data to derive the AUC. This approach allows for the approximation of the integral of time-series air pollution data and provides a robust measure of long-term exposure burden.

The trapezoidal rule sums the areas of trapezoids formed between consecutive daily PM2.5 values, assuming linear changes between each data point. This method is particularly suitable for environmental health studies where pollutant data are collected at regular intervals and sharp fluctuations are common. The AUC thus serves as a proxy for residual or cumulative exposure, capturing the combined intensity and duration of air pollution that participants experienced prior to spirometry testing.

This approach has been previously applied in epidemiological research to estimate pollutant burden and assess its association with chronic respiratory outcomes. For example, Yueming Zhang MMed, et al. (2023) and Valine Atieno Okeyo, et al. (2024) used similar AUC methods to explore long-term air pollution exposure and its health effects in pediatric populations [22,23].

The formula used is:

$$\mathrm{AUC}\;=\;\sum_{i=1}^{n-1}\frac{\left({PM}_{i}+{PM}_{i+1}\right)}{2}\times ∆t$$

where *PM_i_* is the PM2.5 concentration on day*_i_*, and Δ*t* = 1 day.

### 2.3. Research Participants

This study included 110 children (aged 8–12 years) residing in the upper northern region of Thailand with no respiratory symptoms such as asthma. Participants were recruited through convenience sampling from public primary schools in Mueang Chiang Mai District with school and parental consent.

Inclusion criteria were: permanent residence in Chiang Mai, ability to follow spirometry instructions, signed parental/guardian consent, and general good health with no acute illness on the testing day.

Exclusion criteria were: chronic respiratory diseases (e.g., asthma, chronic bronchitis, congenital lung disorders), severe physician-diagnosed chronic conditions (e.g., cardiovascular diseases, kidney failure, infectious diseases, post-surgical conditions, liver diseases, thyroid disorders, thalassemia), recent respiratory tract infection within two weeks, inability or unwillingness to cooperate, conditions affecting cooperation (e.g., mental disorders, substance abuse), lack of consent, withdrawal from the study, or chest pain during testing. These exclusion criteria were applied to minimize potential confounding factors that could independently affect lung function or compromise the validity of spirometry results. Excluding participants unable to cooperate or follow instructions was essential to maintain the accuracy and reproducibility of spirometry measurements, in line with ATS/ERS standards.

#### Sample Size

The sample size for this study was determined using Yamane’s formula [24], which is commonly applied for calculating sample sizes from large populations. The formula is as follows:

$$n=\frac{N}{1+N(e^{2})}$$

where

- n = required sample size
- N = total population in upper northern, Thailand (8–12 year) (65,771)
- e^2^ = margin of error (set at 0.10 or ± 10%)
- Substituting the values into the formula:

$$n=\frac{65,771}{1+65,771\times {\left(0.10\right)}^{2}}=\frac{65,771}{1+657.71}=\frac{65,771}{658.71}\approx 100$$

Thus, the minimum required sample size was calculated to be 100 participants.

Participants aged between 8 and 12 years were selected for this study because this age range represents a critical period of growth and development, particularly in the respiratory system. During this stage, children’s lungs are still maturing, making them more physiologically vulnerable to the adverse health effects of air pollutants such as PM2.5. Furthermore, school-aged children are more likely to engage in outdoor activities, which may increase their exposure to ambient air pollution. Therefore, studying this population provides valuable insights into the early health impacts of air pollution exposure. To account for potential participant dropout, incomplete spirometry measurements, or missing data during the data collection process, the sample size was increased to 110 participants. This adjustment was made to ensure the adequacy and reliability of the data collected, as well as to maintain the statistical power necessary for robust and valid analysis.

### 2.4. Respiratory Symptom Assessment Using the WURSS-K

In this study, the Wisconsin Upper Respiratory Symptom Survey for Kids (WURSS-K) was used as a validated instrument for screening upper respiratory symptoms prior to spirometry testing. The WURSS-K is a 15-item questionnaire that has been validated for measuring symptom severity and quality-of-life impact during acute upper respiratory illness in children. Each item on the WURSS-K is scored using a Likert-type scale ranging from 0 (not at all) to 7 (severe). The survey includes items such as cough, sore throat, runny nose, nasal congestion, sneezing, and headache, along with a general item assessing Overall Health [25].

For the purposes of this study, exclusion criteria were based on the following thresholds: Total Symptom Score ≥ 6: The total score was calculated by summing the scores of all individual symptom items (excluding the Overall Health score). A total score of 6 or more indicated a significant level of respiratory symptom burden.

Any Individual Symptom Score ≥ 2: If a child reported a score of 2 or higher on any single symptom item, this was interpreted as the presence of a moderate or greater symptom that could interfere with accurate spirometry measurement.

Overall, Health Score ≥ 2: If the child rated their overall health as 2 or more (on a 0–7 scale), they were considered to be experiencing a noticeable health disturbance and were excluded from testing to protect participant well-being and data integrity.

Children meeting any one of these criteria were excluded from spirometry testing to ensure that the lung function results were not confounded by acute respiratory symptoms and that the children were physically well enough to participate. This screening approach helped maintain both internal validity of lung function data and ethical standards for participant care. The results of the WURSS-K screening are summarized in Table 2.

### 2.5. Lung Function Testing or Spirometer Testing

Pulmonary function tests (PFTs) were administered to assess lung function among the participating children. The tests adhered to the 2019 standards set forth by the American Thoracic Society (ATS) and the European Respiratory Society (ERS) [26]. A certified pulmonary function specialist, accredited by the Thoracic Society of Thailand under Royal Patronage, conducted the assessments. This study utilized the Contec SP100 spirometer (Contec Medical Systems Co., Ltd., Qinhuangdao, China; 2016). Calibration was performed using a standard calibration syringe (e.g., 3 L). The syringe was securely connected to the device’s mouthpiece, the device was powered on, and approximately three steady injections and withdrawals of air were carried out. The measured values were checked to ensure they closely matched the syringe’s specified volume, with a permissible deviation not exceeding ±3%. If discrepancies were detected, adjustments or professional recalibration were considered. All equipment was cleaned before and after calibration, and daily calibration was conducted in accordance with recommendations for clinical and research applications [24].

#### 2.5.1. Instructions for Pulmonary Function Testing

Prior to conducting the pulmonary function test, each participant received a comprehensive explanation and demonstration of the procedure using age-appropriate language to ensure full understanding. This approach aligns with best practices in spirometry testing, emphasizing the importance of participant comprehension to obtain accurate results. In addition to standard pre-test inquiries, detailed household and lifestyle-related information was collected using a structured Case Record Form (CRF). The CRF included questions on indoor pollution sources (e.g., household smoking, use of air purifiers, and cooking practices) and other environmental or behavioral factors that may influence respiratory health. Participants were also asked about recent medication usage, including bronchodilators or β-blockers, as these can influence test outcomes. Additionally, information regarding their most recent meal was collected, given that heavy meals may affect test performance and impose certain restrictions. Participants were advised to avoid wearing tight or restrictive clothing that could interfere with the test. These considerations, together with the CRF data, are crucial for ensuring the accuracy and reliability of spirometry measurements [27].

The following demographic and anthropometric data were recorded in the spirometry software: weight, height, chest, age, sex, and blood pressure value. Accurate recording of these parameters is essential, as they are used to calculate predicted normal values for lung function parameters, facilitating the assessment of deviations from expected results.

Participants were then instructed to perform the following steps [26]:

1. Take a deep inhalation to fill the lungs completely.
2. Use a nose clip to prevent nasal airflow.
3. Place a disposable mouthpiece securely between the lips to avoid air leakage.
4. Exhale forcefully and rapidly until the lungs felt entirely empty.

#### 2.5.2. Test Administration

To prevent fatigue, participants were limited to a maximum of eight test attempts, with adequate rest periods between trials. The following lung function parameters were measured, and Within-test variability was evaluated based on the 2019 ATS/ERS criteria, requiring that the two best FVC and FEV_1_ values differ by no more than 150 mL Only tests meeting these standards were included in the analysis [28,29]:

1. Forced Vital Capacity (FVC): The total volume of air that can be forcibly exhaled after full inhalation.
2. Forced Expiratory Volume in one second (FEV_1_): The volume of air expelled during the first second of the FVC maneuver.

#### 2.5.3. The Siriraj Equations

The Siriraj equations were specifically developed for use in the Thai population, with height and age as key variables [28,29]. Although subgroup-specific validations in the 8–12-year age group are limited, these equations are widely applied in both clinical and research contexts in Thailand and are considered the most appropriate reference for the study population.

Forced Vital Capacity (FVC):

Male: FVC = (0.0494 × height) − (0.0212 × age) − 3.59 (1)

Female: FVC = (0.0429 × height) − (0.0218 × age) − 2.63 (2)

Forced Expiratory Volume in one second (FEV_1_):

Male: FEV_1_ = (0.0441 × height) − (0.0188 × age) − 2.87 (3)

Female: FEV_1_ = (0.0342 × height) − (0.0205 × age) − 1.58 (4)

Peak Expiratory Flow (PEF):

Male: PEF = (0.0135 × height) − (0.0145 × age) + 8.56 (5)

Female: PEF = (0.0141 × height) − (0.0154 × age) + 6.18 (6)

where

- Height is measured in centimeters (cm)
- Age is measured in years

Calculating % Predicted Values:

Once the predicted PEF is determined, the percentage of predicted PEF (%PEF Prediction) can be calculated using the following formula:

(7) $$\%\mathrm{PEF}\;\mathrm{Prediction}\;=\;(\frac{Measured}{Predicted})\times 100$$

#### 2.5.4. The Adjusted Odds Ratio (AOR)

The Adjusted Odds Ratio (AOR) is a statistical measure commonly used in clinical and epidemiological research to estimate the strength of association between a categorical independent variable (e.g., sex) and a binary outcome (e.g., abnormal lung function), while controlling for potential confounding variables (e.g., BMI). In this study, the AOR was calculated using multivariate logistic regression to assess whether the likelihood of abnormal pulmonary function differed significantly between male and female participants after adjusting for BMI [30,31].

The logistic regression model used was as follows:

(8) $$\mathrm{In}\;(\frac{p}{1-p})\;=\;\beta_{0}\;+\;\beta_{1}\;\mathrm{Sex}\;+\;\beta_{2}\;{\mathrm{BMI}}_{\mathrm{Underweight}}\;+\;\beta_{3}\;{\mathrm{BMI}}_{\mathrm{Overweight}}\;+\;\beta_{4}\;{\mathrm{BMI}}_{\mathrm{Obese}}$$

where

- p = probability of abnormal pulmonary function
- β_0_ = intercept
- β_1_ = coefficient for sex (male vs. female)
- β_2_, β_3_, β_4_ = coefficients for BMI categories (with normal BMI as the reference group)

Since all participants were exposed to similar levels of PM2.5 in the same environment, the analysis did not compare exposure versus non-exposure but rather focused on the association between sex, BMI categories, and pulmonary function outcomes.

### 2.6. Statistical Analysis

In this study, the collected data underwent comprehensive statistical analysis to ensure accuracy and meaningful interpretation. Descriptive statistics were used to summarize the data, including the calculation of means and standard deviations (mean ± SD, 95% confidence intervals) for normally distributed variables, as well as medians and ranges (median, range) for non-normally distributed variables. The prevalence of specific characteristics or conditions within the study population was expressed as percentages and ratios, providing a clear understanding of their distribution [30].

Statistical analyses were performed using IBM SPSS Statistics version 29.0, with a significance level of *p* < 0.05 considered statistically significant. For inferential statistics, appropriate tests were selected based on the nature of the data. The student’s paired *t*-test was used to compare mean differences between selected subgroups. Although the compared groups were not matched individuals in a strict sense, they were drawn from the same school environment, shared similar demographic and exposure characteristics, and were tested under the same protocols and conditions. This quasi-paired structure helped reduce inter-group variability and improve sensitivity in detecting differences. The use of the paired *t*-test in this context was deemed appropriate given the exploratory nature of the study. Additionally, logistic regression analysis was conducted to estimate adjusted odds ratios (AORs), controlling for BMI categorized into four groups: underweight, normal, overweight, and obese [31,32].

## 3. Results

### 3.1. Cumulative PM2.5 Exposure Prior to Lung Function Testing

**Figure 2:**
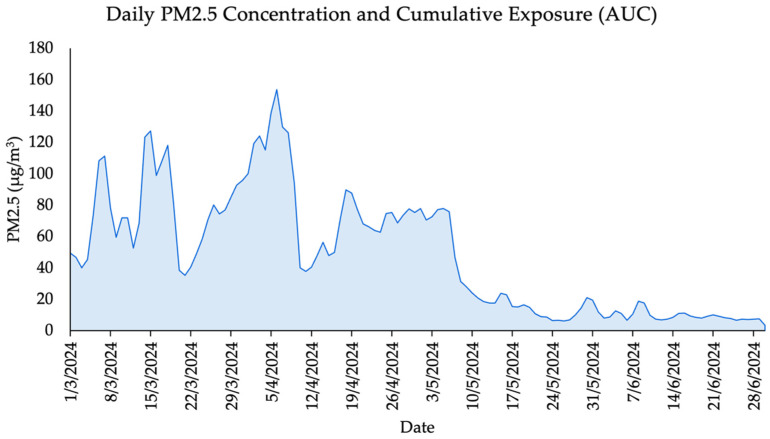
Daily PM2.5 Concentrations and Cumulative Exposure (AUC) Over a 120-Day Period.

The calculated AUC for PM2.5 exposure over the 120-day period prior to lung function assessment was 5941.05 µg/m^3^ × day (Figure 2). This value reflects a substantial cumulative inhalation burden in the study population, particularly during the peak haze season in March and April. Such sustained exposure levels are known to contribute to subclinical and clinical impairments in pulmonary function, especially in children whose lungs are still developing. The observed AUC supports the hypothesis that long-term exposure, rather than acute exposure alone, may play a significant role in the lung function patterns identified in this study.

### 3.2. Screening Results: Respiratory Symptoms by Gender

Table 3 presents the distribution of participants by gender, symptom type, and symptom severity based on WURSS-K scores. Symptoms were categorized as significant if the score was greater than or equal 2. The table includes the number and percentage of children exhibiting either runny nose or cough with a score above the threshold, as well as the number of children who had no significant symptoms. Percentages were calculated based on the total sample size (*n* = 110).

Among the 110 child participants, 58 were male and 52 were female. A total of 3 children (2.7%) were excluded based on WURSS-K screening criteria. Specifically, all exclusions were due to an individual symptom score ≥2, with two children reporting a score of 2 on runny nose and one reporting a score of 2 on cough. All three cases occurred in male participants. No participants met the exclusion thresholds for total symptom score (≥6) or overall health score (≥2). These results indicate that the prevalence of significant respiratory symptoms was low across the sample, and the majority of children were in suitable health to proceed with spirometry testing.

Although the number of children exhibiting significant symptoms was small (2.7%), the fact that all symptomatic cases were male may warrant further investigation. Previous research has suggested that gender differences may influence susceptibility to respiratory symptoms, potentially due to variations in immune responses, airway anatomy, or environmental exposure patterns (e.g., time spent outdoors or involvement in activities that increase pollutant exposure) [33]. However, given the limited number of symptomatic cases in this study, no definitive conclusions can be drawn regarding gender-based susceptibility. Overall, the symptom screening process served not only as a protective measure for ensuring the quality of spirometry data but also provided an early health indicator that may be useful for longitudinal tracking in future studies on respiratory health and air pollution exposure.

### 3.3. The Characteristics of the Participants Who Underwent Spirometry Testing

**Figure 3:**
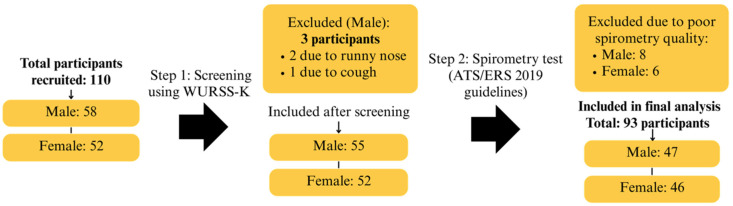
Participant Flow Diagram Showing Enrollment, Screening, Exclusions, and Final Analytical Sample Based on ATS/ERS Criteria.

Of the 110 initial research participants, 17 were excluded for various reasons: 3 due to acute respiratory symptoms, and 14 because their spirometry tests did not meet the within-test variability threshold (≤150 mL difference between the best two FVC and FEV_1_ values), as specified by the 2019 ATS/ERS guidelines [26]. As a result, 93 participants (47 males, 50.54% and 46 females, 49.46%), aged 8–12 years, successfully completed the study, as presented in Figure 3. The average body weight of participants was approximately 40 kg, with a mean height of 143 cm and an average chest circumference of 70.4 cm. All participants were residents of Chiang Mai Province. The physical characteristics of these participants are summarized in Table 4.

### 3.4. PM2.5 on Lung Volume and Lung Capacity

The study found that participants exposed to haze levels exceeding safety standards for a prolonged period had significantly lower FVC values compared to normal reference values (2.16 ± 0.49 L vs. 3.09 ± 0.47 L, respectively). This suggests a reduction in lung volume relative to the expected values for Thai children of the same age. Similarly, both FEV_1_ and PEF were below normal reference values, measuring 1.82 ± 0.42 L and 3.85 ± 1.00 L/s, respectively. These findings indicate a reduced capacity for air ventilation during exhalation. Although the FEV_1_/FVC ratio showed a statistically significant difference (*p* < 0.001) compared to predicted values, the mean value (86.30 ± 13.07%) remained within the Thai reference range, suggesting no clinically significant airway obstruction, as shown in Table 5.

### 3.5. The Number of Respiratory Abnormalities in Individuals with Reduced Lung Volumes

Pulmonary function testing using spirometry plays a crucial role in diagnosing respiratory diseases. In this study, lung function abnormalities were classified based on the Global Lung Function Initiative (GLI 2012) reference equations, which account for age, sex, height, and ethnicity, into the following categories [34]:

Obstructive pattern: Defined as an FEV_1_/FVC ratio below the Lower Limit of Normal (LLN) according to GLI 2012 reference equations for age, sex, height, and ethnicity, indicating airflow limitation due to narrowed or obstructed airways, commonly seen in conditions such as asthma.

Restrictive pattern: Defined by a normal or elevated FEV_1_/FVC ratio (≥LLN) accompanied by a reduced FVC below the Lower Limit of Normal (LLN) based on GLI 2012 reference equations, suggesting a limitation in lung expansion.

Normal spirometry: Defined as both FEV_1_ and FVC at or above the Lower Limit of Normal (LLN), and an FEV_1_/FVC ratio at or above LLN, indicating no significant obstructive or restrictive ventilatory defect.

These definitions were used to classify participants into obstruction, restriction, and normal spirometry groups for analysis. The prevalence and severity distribution of each type of respiratory abnormality are summarized in Table 6.

The most prevalent respiratory abnormality identified was restrictive lung disease, affecting 48 out of 93 participants (51.61%), comprising 17 males and 31 females. The majority of these cases were classified as moderate. The second most common abnormality was obstructive lung disease, observed in 17 participants (18.28%), including 2 males and 15 females, with most also falling under the moderately severe category. Meanwhile, 28 participants (30.11%) demonstrated normal spirometry results. Notably, no cases of mixed defects were identified in this cohort. Additionally, the interpretation of sex differences in restrictive and obstructive spirometry patterns should be approached with caution. The small sample size and uneven distribution between male and female participants limit the generalizability of these findings. Further studies with larger and more balanced cohorts are needed to validate these observations.

The predominance of moderate classifications in restrictive groups raises important clinical concerns. In children, moderately severe restrictive defects suggest a substantial reduction in lung volume, which may impair exercise tolerance, limit daily activities, and, if left unaddressed, could hinder normal lung development, potentially increasing the risk of chronic respiratory diseases in adulthood. Similarly, although less common, moderately severe obstructive abnormalities warrant clinical attention, as they may indicate underlying airway hyperresponsiveness or undiagnosed asthma requiring follow-up care. These findings highlight the importance of routine respiratory surveillance and early interventions to safeguard long-term respiratory health in this vulnerable pediatric population [35].

### 3.6. Factors Associated with Abnormal Pulmonary Function Following PM2.5 Exposure

Table 7 presents logistic regression analysis was conducted to estimate the adjusted odds ratio (AOR), controlling for BMI categorized into four groups: underweight, normal, overweight, and obese. The results showed that males had a significantly lower likelihood of abnormal pulmonary function compared to females (AOR = 0.084; 95% CI: 0.017–0.417; *p* = 0.002) after adjusting for BMI. In contrast, the BMI categories showed no statistically significant associations when compared with the normal BMI group: underweight (AOR = 0.99; 95% CI: 0.27–3.60; *p* = 0.99), overweight (AOR = 1.30; 95% CI: 0.18–9.67; *p* = 0.80), and obese (AOR = 4.84; 95% CI: 0.22–104.82; *p* = 0.32). Importantly, the small sample sizes, particularly in the obese group, may have contributed to the wide confidence intervals and reduced the precision of the risk estimates.

This pronounced sex difference highlights the crucial role of biological sex as an independent risk or protective factor for pulmonary health. Several possible mechanisms could explain why females appear more vulnerable to abnormal pulmonary function. Biologically, females typically have smaller lung volumes and lower respiratory reserves compared to males, limiting their compensatory capacity during respiratory stress. Hormonal factors, such as the pro-inflammatory effects of estrogen, may amplify susceptibility to pollutants or airway irritants [36].

## 4. Discussion

This study highlights the adverse impact of prolonged PM2.5 exposure on the respiratory health of primary school children in Chiang Mai Province. The predominance of restrictive over obstructive lung patterns suggests that chronic exposure may contribute to reduced lung volume, likely due to alveolar inflammation or early fibrotic changes rather than airway narrowing. While previous studies often emphasize obstructive outcomes such as asthma, the current findings align with emerging evidence of interstitial lung alterations and impaired pulmonary development in children chronically exposed to air pollution [37,38].

Although spirometry was conducted during a low-pollution period (July), cumulative exposure was estimated using the area under the curve (AUC) of daily PM2.5 concentrations over the preceding 120 days. This approach captures residual or subacute exposure, rather than immediate effects, and provides insight into the extended burden of pollution. The elevated AUC values observed in this cohort reflect substantial and repeated seasonal exposure particularly during the March–April haze period which may have led to persistent impairments in lung structure and function.

Such cumulative exposure likely exerts progressive impacts on lung parenchymal development, supporting the hypothesis of delayed or ongoing injury that persists beyond the pollution event. The predominance of restrictive patterns may indicate underlying structural changes such as alveolar simplification or early interstitial remodeling, which are consistent with chronic, rather than acute, pulmonary responses. These patterns may emerge gradually over repeated exposure cycles, particularly during sensitive periods of lung growth in childhood.

Testing during a cleaner air season may have further minimized the confounding influence of acute exposure, allowing underlying chronic or residual impairments to be more readily detected. The divergence between these findings and studies highlighting obstructive outcomes may also be explained by differences in study populations. Whereas prior research often focused on clinical subgroups, such as children with asthma, this study involved a general school population without pre-existing respiratory diagnoses—potentially capturing earlier, subclinical restrictive changes that are underrepresented in asthma-focused cohorts.

Interestingly, no participants exhibited a mixed ventilatory defect defined as concurrent obstruction and restriction. Several factors may explain this absence. First, the generally healthy profile of participants, all without physician-diagnosed chronic respiratory diseases, likely reduced the probability of detecting complex impairments. Mixed patterns are more often observed in individuals with advanced or poorly controlled conditions, such as severe asthma with airway remodeling, cystic fibrosis, or post-infectious sequelae. Second, testing was conducted outside the peak PM2.5 season, lowering the likelihood of capturing acute bronchoconstriction superimposed on chronic restriction. Third, our use of the GLI 2012 LLN classification, which clearly defines and separates obstruction and restriction, may have reduced classification overlap and therefore the probability of identifying mixed patterns compared to fixed-ratio methods. This suggests that classification thresholds could, in part, explain the absence of mixed findings. Finally, potential measurement bias cannot be excluded, as our protocol did not include more sensitive lung volume or gas exchange assessments (e.g., body plethysmography or diffusion capacity testing), which could detect early or subclinical mixed patterns.

Importantly, a significant gender disparity was observed: females demonstrated a higher prevalence and severity of respiratory abnormalities compared to males. This disparity likely reflects an interplay of biological, anatomical, physiological, behavioral, and social factors. Anatomically, females generally have smaller airway diameters relative to lung size, which may predispose them to greater airflow limitation when exposed to environmental pollutants. Physiologically, heightened inflammatory responses and differences in pulmonary immune regulation have been suggested, with estrogen potentially amplifying oxidative stress pathways, thereby increasing vulnerability to particulate matter exposure [39,40,41]. Behavioral and social factors may further compound this risk. For example, differences in outdoor activity patterns, time spent in indoor environments with varying air quality could contribute to observed disparities. Although females showed higher rates of respiratory impairments, the underlying mechanisms remain speculative due to the absence of hormonal or biomarker data. Thus, biological explanations should be interpreted with caution. Future research should adopt a multidimensional approach, integrating biological determinants (such as hormonal profiles, airway anatomy, and immune responses) alongside behavioral and social dimensions, to comprehensively investigate sex-specific susceptibilities to air pollution. Such an approach will be critical for informing tailored public health interventions and risk mitigation strategies.

Several limitations must be acknowledged. First, the study lacked a low-exposure control group, which limited comparisons across different exposure levels.

Second, PM2.5 exposure was estimated using data from a single fixed-site monitoring station (36T). While this approach provides a general estimate of ambient exposure, it may not reflect individual-level variation, particularly due to differences in time spent indoors, proximity to pollution sources, or use of air filtration. Although personal exposure monitoring was not conducted, contextual data were collected through structured questionnaires, which included household and lifestyle factors such as cooking fuels, ventilation practices, incense or mosquito coil use, and tobacco smoke exposure.

These indoor sources are known contributors to fine particulate matter, and their effects may have confounded the observed associations. However, these variables were not included as covariates in the final statistical models, so residual confounding from unmeasured or underreported indoor pollutants cannot be ruled out. Future studies should incorporate these variables into multivariable models and consider quantitative measurements of indoor air pollutants to better isolate the effects of ambient PM2.5.

Third, the cumulative PM2.5 exposure was estimated indirectly using the area under the curve (AUC) over 120 days, rather than through direct personal monitoring or long-term follow-up. The cross-sectional nature of the study also limits causal inference and prevents assessment of exposure-response trajectories over time. Additionally, possible subclinical respiratory infections without overt symptoms may have influenced spirometry results but were not assessed.

Fourth, spirometry interpretation relied on a hybrid reference approach: GLI 2012 equations were used to classify patterns, while percent predicted values were calculated using the Siriraj equations. This was necessary due to the absence of LLN thresholds in Thai pediatric references but may affect consistency. A sensitivity analysis revealed that reference equation choice significantly influenced pattern classification, reinforcing the need for population-specific standards.

Lastly, while sex differences in lung function were explored, the absence of hormonal or biomarker data limits biological interpretations. The observed associations, particularly those involving sex-based disparities, should therefore be viewed as preliminary and interpreted with caution.

## 5. Conclusions

In a school-based sample of 93 children aged 8–12 years with acceptable spirometry, 52% demonstrated restrictive patterns, 18% obstructive patterns, and 30% normal function. The mean FEV_1_/FVC ratio was 86.30 ± 13.07% (within normal limits), whereas mean percent-predicted FVC, FEV_1_, and PEF were significantly below predicted, indicating that restriction was the dominant pattern. After adjustment for BMI, males had substantially lower odds of any pulmonary abnormality than females (AOR 0.084; 95% CI 0.017–0.417; *p* = 0.002), suggesting sex-based differences in susceptibility. Exposure was assigned from a single fixed-site monitor within 2 km of the test site and from one season, which may not capture spatial or longer-term variability; indoor exposures were not directly measured. Even with these limitations, the quantitative patterns are consistent with reduced lung volumes in this age group under haze-related PM2.5 exposure. Public-health implications include strengthening seasonal air-quality protections, reducing indoor exposure in schools and homes (e.g., filtration), and prioritizing communication for vulnerable groups. Future studies should employ longitudinal, multi-season designs with personal and indoor monitoring and evaluate additional clinical indices (e.g., respiratory muscle strength) to clarify mechanisms underlying the observed sex differences and to better safeguard children’s respiratory health.

## Figures and Tables

**Table 1:** The mean PM2.5 concentration from the air quality monitoring station in Mueang District, Chiang Mai Province (36T) during January–December 2024 [21].

Month	Concentration of PM2.5 (36T)			
	Average PM2.5 24 h. (µg/m^3^)			Average Monthly
	Max	Min	Day > std.	
January	38.4	15.0	1/31	24
February	54.2	14.7	8/29	34
March	152.7	29.8	30/31	74
April	163.9	28.1	28/30	82
May	89.8	4.6	7/31	29
June	23.0	6.2	0/30	9
July	15.0	6.1	0/31	9
August	13.7	6.1	0/31	9
September	12.8	6.6	0/30	9
October	23.3	6.7	0/31	14
November	22.3	5.8	0/30	17
December	29.8	6.9	0/31	22

Standard (std.): The 24-h average standard for PM2.5 in Thailand at 37.5 µg/m^3^.

**Table 2:** Screening criteria for spirometry using WURSS-K questionnaire.

Assessment Item	Screening Criteria	Result	Notes
Symptom Score	<6 points	Pass	Indicates mild symptoms only
	≥6 points	Fail	Indicates moderate to severe respiratory symptoms
Individual Symptom	<2 points	Pass	No single symptom is moderate or severe
	≥2 points in any item	Fail	Presence of a prominent symptom
Overall, Health	<2 points	Pass	The child reports feeling generally well
	≥2 points	Fail	The child reports feeling moderately to severely unwell

A participant will be classified as Fail if any one of the three criteria is met.

**Table 3:** Distribution of respiratory symptoms by gender, symptom type, and severity based on WURSS-K scores (*n* = 110).

Gender	Total (*n*)	Symptom Type	Number with Symptom ≥ 2	Percentage of Total (%)	Number Without Symptom ≥ 2
Male	58	Runny nose	2	1.8	55
		Cough	1	0.9	
Female	52	Runny nose	0	0.0	52
		Cough	0	0.0	
Total	110		3	2.7	107

Symptom ≥ 2 refers to a score greater than or equal 2 on the WURSS-K for each respective symptom.

**Table 4:** Characteristics of the study subjects who underwent spirometry testing.

Baseline Characteristics	Total (*n* = 107)	Success Group (*n* = 93)	Failure Group (*n* = 14)
Age (years)	107 (100.00)	93 (86.90)	14 (13.10)
8 years	2 (1.86)	0 (0.00)	2 (100.00)
9 years	20 (18.69)	18 (90.00)	2 (10.00)
10 years	27 (25.23)	23 (85.18)	4 (14.81)
11 years	40 (37.38)	35 (87.50)	5 (12.50)
12 years	18 (16.82)	17 (94.44)	1 (5.55)
Gender, Male	55 (51.40)	47 (50.54)	8 (14.54)
Gender, Female	52 (48.59)	46 (49.56)	6 (11.54)
Weight (kg)	40.23 ± 9.48	39.83 ± 12.05	42.84 ± 17.64
Height (cm)	143.07 ±1 2.95	143.25 ± 9.27	141.82 ± 10.65
BMI (kg/m^2^)	19.31 ± 4.67	19.14 ± 4.44	20.47 ± 5.82
SBP (mmHg)	108.23 ± 10.78	107.97 ± 10.56	110 ± 11.98
DBP (mmHg)	70.32 ± 7.39	70.29 ± 7.38	70.57 ± 7.45
Heart rate (bpm)	91.45 ± 12.37	91.01 ± 12.62	94.43 ± 10.08
Chest (cm)	70.41 ± 11.14	70.06 ± 10.43	72.79 ± 14.83

Values are presented as numbers (*n*, %) or mean ± standard deviation (Mean ± SD). BMI: Body Mass Index; SBP: Systolic Blood Pressure; DBP: Diastolic Blood Pressure.

**Table 5:** The mean ± standard deviation (mean ± SD) and 95% confidence interval of lung volume and pulmonary function test (PFT) results were analyzed in 93 healthy volunteers exposed to PM2.5.

	PFT_PM2.5_	PFT_Predicted_	PFT_PM2.5% of Prediction (%)_	*p*-Value
FVC	2.16 ± 0.49	3.09 ± 0.47	70.33 ± 12.89	<0.001
FEV_1_	1.82 ± 0.42	3.18 ± 0.38	57.05 ± 8.40	<0.001
FEV_1_/FVC	84.65 ± 7.42	99.69 ± 12.80	86.30 ± 13.07	<0.001
PEF	3.85 ± 1.00	9.20 ± 1.15	42.27 ± 11.27	<0.001

FVC: Forced Vital Capacity; FEV_1_: Forced Expiratory Volume in one second; PEF: Peak Expiratory Flow.

**Table 6:** Frequency (*n*) and percentage (%) distribution of obstruction, restriction, mixed defect, and normal spirometry among 93 volunteers.

Severity Level		Obstruction	Restriction	Mixed Defect	Normal Spirometry
	Total, *n* (%)	17 (18.28)	48 (51.61)	0	28 (30.11)
Mild, *n* (%)	Male	0	0		
	Female	0	2 (2.15)		
Moderate, *n* (%)	Male	0	10 (10.75)		
	Female	3 (3.23)	13 (13.98)		
Moderately severe, *n* (%)	Male	1 (1.08)	5 (5.38)		
	Female	8 (8.6)	7 (7.53)		
Severe, *n* (%)	Male	1 (1.08)	2 (2.15)		
	Female	4 (4.30)	9 (9.68)		
Very severe, *n* (%)	Male	0	0		
	Female	0	0		

Patterns classified by GLI 2012 LLN; severity graded by FEV_1_%pred (Siriraj reference equations).

**Table 7:** Association between sex, BMI categories, and abnormal pulmonary function among study participants (*n* = 93).

Variable	Adjusted Odds Ratio (AOR)	95% Cl	*p*-Value
Sex (Male vs. Female)	0.084	0.017–0.417	0.002 *
Underweight (vs. Normal)	0.99	0.27–3.60	0.99
Overweight (vs. Normal)	1.3	0.18–9.67	0.8
Obese (vs. Normal)	4.84	0.22–104.82	0.32

* Statistically significant (*p* < 0.05).

## Data Availability

The data presented in this study are available on request.

## Abbreviations

The following abbreviations are used in this manuscript:

ATS	American Thoracic Society
COPD	Chronic Obstructive Pulmonary Disease
ERS	European Respiratory Society
FEV1	Forced Expiratory Volume in one second
FVC	Forced Vital Capacity
AOR	Adjusted Odds Ratio
PEF	Peak Expiratory Flow
PFTs	Pulmonary Function Tests
PM2.5	Fine Particulate Matter
RIHES	Research Institute for Health Sciences
WHO	World Health Organization
